# HDAC Inhibition for Optimized Cellular Immunotherapy of NY-ESO-1-Positive Soft Tissue Sarcoma

**DOI:** 10.3390/biomedicines10020373

**Published:** 2022-02-03

**Authors:** Wenjie Gong, Lei Wang, Maria-Luisa Schubert, Christian Kleist, Brigitte Neuber, Sanmei Wang, Mingya Yang, Angela Hückelhoven-Krauss, Depei Wu, Anita Schmitt, Carsten Müller-Tidow, Hiroshi Shiku, Michael Schmitt, Leopold Sellner

**Affiliations:** 1 Department of Internal Medicine V, Heidelberg University Hospital, 69120 Heidelberg, Germany; Wang.Lei@med.uni-heidelberg.de (L.W.); Maria-Luisa.Schubert@med.uni-heidelberg.de (M.-L.S.); Brigitte.Neuber@med.uni-heidelberg.de (B.N.); Sanmei.Wang@med.uni-heidelberg.de (S.W.); Mingya.Yang@med.uni-heidelberg.de (M.Y.); Angela.Hueckelhoven@med.uni-heidelberg.de (A.H.-K.); anita.schmitt@med.uni-heidelberg.de (A.S.); carsten.mueller-tidow@med.uni-heidelberg.de (C.M.-T.); michael.schmitt@med.uni-heidelberg.de (M.S.); 2 Department of Hematology, First Affiliated Hospital of Soochow University, Suzhou 215005, China; wudepei@suda.edu.cn; 3 Department of Nuclear Medicine, Heidelberg University Hospital, 69120 Heidelberg, Germany; christian.kleist@med.uni-heidelberg.de; 4 Department of Hematology, First Affiliated Hospital of Nanjing Medical University, Nanjing 210002, China; 5 German Cancer Consortium (DKTK), National Center for Tumor Diseases (NCT), 69120 Heidelberg, Germany; 6 Department of Immuno-Gene Therapy, Mie University, Tsu 514-8507, Japan; shiku@clin.medic.mie-u.ac.jp; 7 Takeda Pharmaceuticals, Cambridge, MA 02139, USA

**Keywords:** HDAC inhibition, panobinostat, vorinostat, NY-ESO-1-specific T cells, soft tissue sarcoma

## Abstract

Adoptive cell therapy with NY-ESO-1-specific T cells is a promising option for the treatment of soft tissue sarcoma (STS) but achieves only transient tumor control in the majority of cases. A strategy to optimize this cell therapeutic approach might be the modulation of the expression of the cancer-testis antigen NY-ESO-1 using histone deacetylase inhibitors (HDACis). In this study, the ex vivo effect of combining NY-ESO-1-specific T cells with the clinically approved pan HDACis panobinostat or vorionstat was investigated. Our data demonstrated that STS cells were sensitive to HDACis. Administration of HDACi prior to NY-ESO-1-specific T cells exerted enhanced lysis against the NY-ESO-1+ STS cell line SW982. This correlated with an increase in the NY-ESO-1 and HLA-ABC expression of SW982 cells, as well as increased CD25 expression on NY-ESO-1-specific T cells. Furthermore, the immune reactivity of NY-ESO-1-specific CD8+ T cells in terms of cytokine release was enhanced by HDACis. In summary, pretreatment with HDACis represents a potential means of enhancing the cytotoxic efficacy of NY-ESO-1-specific T cells against NY-ESO-1-positive STS.

## 1. Introduction

Soft tissue sarcoma (STS) is a rare heterogeneous disease originating from mesenchyme comprising more than 70 subtypes [1]. Surgical resection, either alone or in combination with radio- or chemotherapy, is the standard treatment for patients with localized lesions. For metastatic patients, systemic therapy including chemotherapeutic and novel biological compounds such as tyrosine-kinase inhibitors and monoclonal antibodies currently plays the most relevant role in the management of STS [2]. However, the benefits of these treatment approaches are limited in the advanced setting [3]. Therefore, new strategies are necessary to improve the clinical outcome in STS patients.

The cancer-testis antigen NY-ESO-1 is an ideal target for the adoptive cell transfer (ACT) of tumor-specific T cells. Besides on tumor cells, including STS, glioma, mesothelioma and myelomas [4], it is exclusively expressed in adults on germ line cells that lack human leucocyte antigen (HLA) molecules for T cell recognition [5,6]. NY-ESO-1 is believed to be an immunogenic protein, as it has been shown to elicit a spontaneous humoral and cellular immune response in patients [7]. Clinical trials with genetically modified NY-ESO-1-specific T cells displayed encouraging anti-tumor activity in STS [8,9,10]. However, ACT with NY-ESO-1-specific T cells can often only achieve transient tumor control [10], pointing to a strong medical need to optimize immunotherapies using NY-ESO-1-specific T cells.

Malignant tumors have diverse phenotypic and molecular characteristics at the intratumor level. Intratumor heterogeneity (ITH) refers to distinct tumor cell populations (with different molecular and phenotypical profiles) within the same tumor specimen [11]. It is a typical characteristic of solid tumors that can also affect tumor-associated antigens (TAAs) and has been correlated with shorter overall survival (OS). A higher neoantigen burden and a lower ITH appeared related to improved outcome and durable clinical benefit [12]. Consequently, the pharmacologic reduction of ITH and thereby modulation of TAA might lead to an improvement in therapeutic efficacy. The discovery of the epigenetic silencing of TAAs and their potential restoration using epigenetic drugs lead to an augmented response to immunotherapy [13]. Therefore, the epigenetic regulation of NY-ESO-1 may be a promising approach to improve ACT. Epigenetic mechanisms such as DNA methylation as well as histone acetylation and methylation play a crucial role to regulate gene activity. They are major determinants in the epigenetic silencing of genes. Cartron et al. elucidated the molecular mechanisms and factors involved in the epigenetic regulation of the NY-ESO1 gene promoter and demonstrated that histone deacetylase 1 (HDAC1), a member of the HDAC family, negatively regulates the expression of NY-ESO-1 [14]. The FDA approved the pan-histone deacetylase inhibitors (HDACis) vorinostat and panobinostat that can both inhibit HDAC1. Vorinostat is approved for the treatment of patients with relapsed/refractory (r/r) cutaneous T-cell lymphoma [15], whereas panobinostat is approved for the treatment of r/r multiple myeloma (MM) [16,17,18].

In this study, the two pan-HDACis, panobinostat and vorinostat, were combined with NY-ESO-1-specific T cells to improve anti-tumor efficacy in STS. First, the two HDACis were titrated on STS cell lines to evaluate cytotoxicity and optimal dose for subsequent experiments. Then, HDACi-mediated changes in NY-ESO-1 expression on STS cells were determined by qRT-PCR and Western blot. In addition, the impact of HDACi pretreatment on NY-ESO-1-specific T cell cytotoxicity was investigated. Furthermore, the HDACi-mediated changes of additional surface markers on STS cells including the immune checkpoints CD80 and programmed cell death protein ligand 1 (PD-L1), MHC class 1 HLA-ABC, as well as the T cell activation marker CD25, were investigated by flow cytometry. Finally, differences in the intracellular cytokine production of NY-ESO-1-specific T cells activated by STS cells with and without HDACi pretreatment were evaluated.

## 2. Materials and Methods

### 2.1. Primary Cells

Peripheral blood samples from buffy coats were obtained from healthy donors (HDs) at the Heidelberg University Hospital (Heidelberg, Germany). Peripheral blood mononuclear cells (PBMCs) were purified using a FicoLite-H density gradient (Linaris, Dossenheim, Germany) and cryopreserved until usage. The study was approved by the Ethics Committee of the University of Heidelberg (S-254/2016; 16 June 2016) and conducted in accordance with the declaration of Helsinki.

### 2.2. Cell Lines

The STS cell lines SW982 (NY-ESO-1+ HLA-A2+) and Fuji (NY-ESO-1- HLA-A2+) were kindly provided by Prof. Hiroshi Shiku (Mie University, Tsu, Japan). SW982 cells were cultured in DMEM and Fuji cells were cultured in RPMI 1640 supplemented with 10% heat-inactivated fetal bovine serum (FBS) (all Thermo Fisher Scientific, Waltham, MA, USA).

### 2.3. Compounds

The HDAC inhibitors panobinostat and vorionstat were purchased from Selleck Chemicals (Houston, TX, USA), reconstituted in dimethyl sulfoxide (DMSO, Honeywell, Morristown, NJ, USA) and stored at −80 °C. Thawing and dilution were performed immediately prior to usage.

### 2.4. NY-ESO-1-Specific T Cell Generation

NY-ESO-1-specific T cells were generated as described previously [19,20], using a protocol adapted from a clinical trial (NCT02869217) in Japan. In brief, cryopreserved human PBMCs from HDs were thawed and cultured in GT-T551 medium (Takara Bio, Shiga, Japan) supplemented with 0.6% human serum (ZenBio, Durham, NC, USA) and 2% human serum albumin (US biological, Salem, MA, USA). Day 0 of the production process was defined as the first day of T cell activation. Non-tissue culture-treated 12-well plates (Corning, Corning, NY, USA) were pre-coated with 5 μg/mL anti-CD3 (OKT3; Biolegend, San Diego, CA, USA) and 25 μg/mL retronectin (Takara Bio, Shiga, Japan) one day before T cell activation. On day 0, PBMCs were added to these pre-coated culture plates in culture medium supplemented with 600 U/mL IL-2 (Novartis, Basel, Switzerland). On day 4, 24-well non-tissue culture-treated plates (Corning) were coated with 20 μg/mL of retronectin (Takara Bio) and 3.8 × 10^5^ activated T cells per well were transduced with the MS3II-NY-ESO-1-siTCR retroviral vector (kindly provided by Hiroshi Shiku, Mie University, Tsu, Japan). The plates were centrifuged at 2000× *g*, 32 °C for 2 h during the transduction process. Culture medium changes were scheduled on days 7, 10 and 14 with the new addition of IL-2 (600 U/mL). Cells were counted and cryopreserved on day 15 of the production process.

### 2.5. Resting Procedure for NY-ESO-1-Specific T Cells after Thawing

Genetically modified T cells display decreased functional activity after cryopreservation [21]. To recover T cell functions, a resting step according to our previous study [22] was included, before cellular assays with NY-ESO-1-specific T cells were performed: After thawing and washing, NY-ESO-1-specific T cells were re-suspended at 2 × 10^6^ cells/mL in culture medium supplemented with 600 U/mL IL-2 (Novartis) in a 50 mL tube (Greiner Bio-One, Frickenhausen, Germany). The tube was placed at a slope of 5° above horizon and the cap was unscrewed to allow gas exchange. After an overnight resting period, rested NY-ESO-1-specific T cells were washed, re-counted and used for further experiments.

### 2.6. CellTiter Glo^®^ Cell Viability Assay

The optimal cell number per well was determined by cells in an exponential proliferation phase after 72 h of culture (data not shown). SW982 (1.5 × 10^3^ cells/well), Fuji (2 × 10^3^ cells/well) or NY-ESO-1-specific T (5 × 10^5^ cells/well) cells were seeded in 40 µL medium into 384-well plates (Greiner Bio-One) and incubated overnight. The next day, serial dilutions of panobinostat or vorinostat were added to the pre-seeded cells. After 48 h of treatment, 12 µL of CellTiter-Glo^®^ reagent (Promega, Madison, WI, USA) was added to each well and incubated for 15 min. Luminescence was measured using an EnSight Multimode plate reader (PerkinElmer, Waltham, MA, USA). Data were calculated as relative luminescence compared to the solvent (DMSO) control. The half maximal inhibitory concentrations (IC50) were calculated from relative viability plots of serial dilutions using Graphpad Prism 8.0 software (GraphPad Software, La Jolla, CA, USA). The subscript index indicates the respective assay duration used to calculate the IC50 value (e.g., IC50_48h_ = 48 h). All experiments were performed in duplicates.

### 2.7. qRT-PCR

STS cells (SW982 and Fuji) were seeded in 6-well plates (Sarstedt, Nümbrecht, Germany) and treated with panobinostat or vorinostat for 48 h. After harvesting the cells with trypsin-EDTA (Thermo Fisher Scientific), total RNA was extracted with the Qiagen RNeasy mini kit (Qiagen, Hilden, Germany) and converted to cDNA using the SuperScript III first-strand System (Thermo Fisher Scientific). qRT-PCR was performed in triplicates using the CFX96 TouchTM Real-Time PCR Detection System (Bio-Rad, Berkeley, CA, USA) with specific primer/probe sets and following thermal cycling profile: 50 °C for 2 min, 95 °C for 10 min, 49 cycles at 95 °C for 15 s, and 60 °C for 60 s. The expression of GAPDH was used as an internal standard. All transcript levels were normalized to GAPDH RNA expression as well as to the DMSO control to calculate relative fold induction of expression using the comparative Ct (ΔΔCt) method.

### 2.8. Western Blot

STS cells (SW982 and Fuji) were seeded in T25 flasks (Sarstedt) and treated with panobinostat or vorinostat for 48 h. After detaching using trypsin-EDTA, total cellular proteins were extracted using RIPA buffer (Thermo Fisher Scientific) supplemented with a complete protease inhibitor cocktail (Sigma-Aldrich) for 30 min on ice. Quantification of the extracted proteins was performed by the BCA method using Pierce™ BCA Protein Assay Kit (Thermo Fisher Scientific). Protein was diluted with 4 × loading buffer (Thermo Fisher Scientific) and heated up to 95 °C for 5 min. A total of 10 μg of protein was separated by NuPAGE 4–12% Bis-Tris protein Gel (Thermo Fisher Scientific) and transferred to a nitrocellulose transfer membrane (Thermo Fisher Scientific). Incubation with primary antibodies was performed overnight at 4 °C. Immunoblots were washed with PBST at room temperature and incubated with secondary antibodies for 1 h. Signals were detected using the ECL Prime Western blotting detection reagent (GE Healthcare, Little Chalfont, UK) and visualized by an Amersham Imager 600 (GE Healthcare). Data were analyzed using ImageJ (NIH, Bethesda, MY, USA).

### 2.9. Chromium-51-Release Cytotoxicity Assay

The SW982 target cells were pretreated with panobinostat, vorinostat or DMSO (solvent control) for 48 h. Specific lysis of SW982 cells by NY-ESO-1-specific T cells was evaluated using a 12 h Chromium-51 (^51^Cr) release assay as described previously [19,20]. In brief, pretreated target cells were labeled with ^51^Cr (Hartmann Analytic, Braunschweig, Germany) for 2 h and co-incubated with T cells (effector cells) for 12 h in the absence of HDACi in 96-well U-bottom microplates (Greiner Bio-One) at 37 °C and 5% CO2. An effector to target cell (E:T) ratio of 10:1 i was used. Maximal release was determined by incubating the target cells with 1% Triton X-100 (Merck KGaA, Darmstadt, Germany). Spontaneous release was assessed with medium only. A total of 75 μL of culture supernatant was added to Ultima Gold liquid scintillation cocktail (PerkinElmer, Waltham, MA, USA). Data acquisition was performed on a Wallac Winspectral 1414 liquid scintillation counter (PerkinElmer). All experiments were performed in triplicates. Following formula was used to calculate specific lysis: % specific lysis = (^51^Cr release in the test well—spontaneous ^51^Cr release)/(maximum ^51^Cr release—spontaneous ^51^Cr release) × 100.

### 2.10. Flow Cytometric Analysis of SW982 and NY-ESO-1-Specific T Cells

To exclude dead cells, the Near-IR Dead Cell Stain Kit (Thermo Fisher Scientific) was used. NY-ESO-1-specific TCR expression was evaluated using the PE-conjugated A0201 NY-ESO-1-tetramer (kindly provided by Hiroshi Shiku, Mie University, Tsu, Japan). The following fluorochrome-conjugated antibodies were used for immunophenotyping of different surface markers on SW982 cells as well as NY-ESO-1-specific T cells: anti-CD80-APC (BioLegend, San Diego, CA, USA), anti-HLA-ABC-FITC (eBioscience, San Diego, CA, USA), anti-programmed cell death protein ligand 1 (PD-L1)-PE (eBioscience), anti-CD3-V500 (AmCyan) (BD Biosciences, San Diego, CA, USA), anti-CD8-Parcific blue (BioLegend), anti-CD25-PE-Cy7 (BioLegend), anti-CD4-Alexa Fluor 700 (eBioscience). Data acquisition was performed on an LSR II device (BD Biosciences) and data were analyzed using the FlowJo software (TreeStar, Ashland, OR, USA).

### 2.11. Intracellular Cytokine Staining

SW982 cells were pretreated with panobinostat, vorinostat or DMSO (solvent control) for 48 h. Subsequently, pretreated SW982 cells were co-cultured with NY-ESO-1-specific T cells in the absence of HDACi for 6 h in 96-well U-bottom microplates (Greiner BioOne). For intracellular cytokine retention, the cytokine secretion inhibitor Brefeldin A (BioLegend) was applied. Before fixation and permeabilization using the Foxp3 fix/perm buffer kit (Miltenyi Biotec, Bergisch Gladbach, Germany), cells were stained with Near-IR (Thermo Fisher Scientific), NY-ESO-1-specific TCR and surface markers. Afterwards, intracellular staining was performed with anti-interferon gamma (IFN-γ)-Alexa Fluor 488 (Biolegend) and anti-tumor necrosis factor (TNF)-BV421 (BD Biosciences). Data acquisition was conducted on an LSR II flow cytometer (BD Biosciences). Data were analyzed using the FlowJo software (TreeStar).

### 2.12. Statistical Analysis

Statistical analysis was performed using Excel (Microsoft, Redmond, WA, USA) and Graphpad Prism 8.0 (GraphPad Software, San Diego, CA, USA). *p*-values were calculated using either the parametric two-way student t-test or one-way ANOVA. *p*-values < 0.05 were considered statistically significant. Graphs and tables were designed using Excel, Graphpad Prism 8.0 (GraphPad Software) and PowerPoint (Microsoft). If not otherwise mentioned, results are presented as mean ± standard deviation (SD).

## 3. Results

### 3.1. Panobinostat and Vorinostat Induce Dose-Dependent Cytotoxicity in STS Cell Lines as Well as NY-ESO-1-Specific T Cells

The cytotoxicity of panobinostat and vorinostat was tested on the STS cell lines SW982 and Fuji as well as NY-ESO-1-specific T cells using the CellTiter Glo^®^ cell viability assay. All cells were treated separately with serial dilutions of panobinostat or vorinostat.

**Figure 1:**
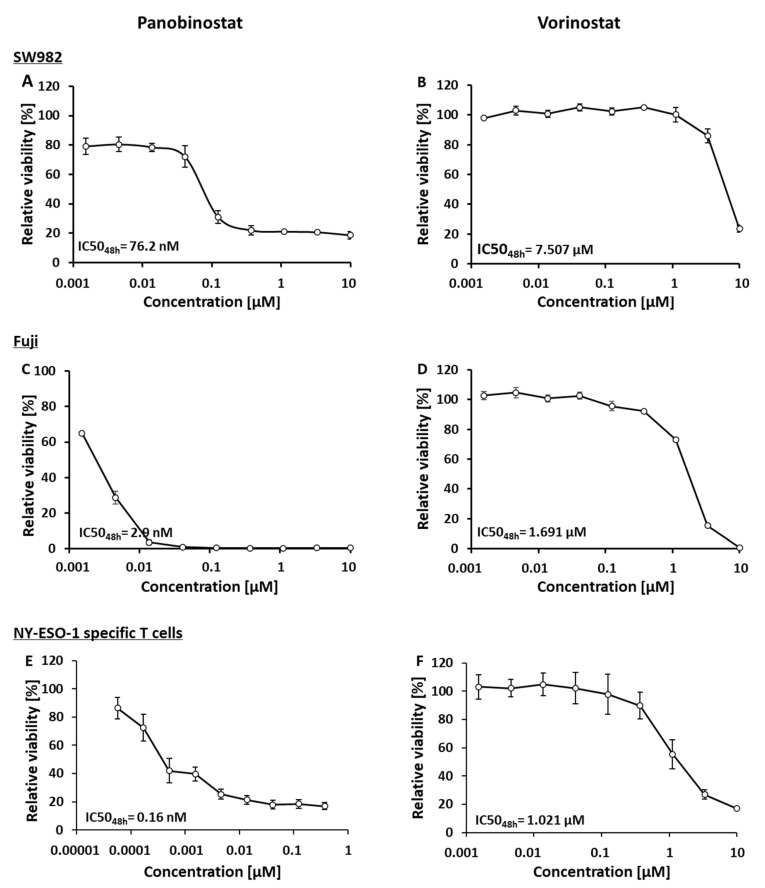
Cytotoxicity of the pan HDAC inhibitors panobinostat and vorinostat on the STS cell lines SW982 (**A**,**B**) and Fuji (**C**,**D**) and NY-ESO-1-specific T cells (**E**,**F**).

Panobinostat and vorinostat were diluted 1:3 from 10 μM to 1.524 nM for SW982 cells and Fuji cells. DMSO served as a solvent control. Both panobinostat and vorinostat influenced the viability of the sarcoma cells in a dose-dependent manner (Figure 1). For SW982 cells, a pronounced loss of viability was observed when the concentration of panobinostat (Figure 1A) or vorinostat (Figure 1B) exceeded 40 nM or 3.33 μM, respectively. The IC50_48h_ values of panobinostat and vorinostat were 76 nM and 7.507 μM, respectively (Figure 1A,B). Fuji cells were even more sensitive to these two HDACis when compared to SW982 cells. The viability of Fuji cells dropped rapidly when the concentration of panobinostat was higher than 1.5 nM (Figure 1C) or the concentration of vorinostat was higher than 1.11 μM (Figure 1D). The corresponding IC50_48h_ of panobinostat and vorinostat were 2.9 nM and 1.691 μM, respectively (Figure 1C,D).

Next, we evaluated the impact of panobinostat and vorinostat on the viability of NY-ESO-1-specific T cells. Due to the above observed high toxicity of panobinostat towards STS cells, we diluted panobinostat 1:3 from 0.370 μM to 0.056 nM for NY-ESO-1-specific T cells, while vorinostat was still diluted 1:3 from 10 μM to 1.524 nM. Panobinostat and vorinostat exerted dose-dependent effects on the viability of NY-ESO-1-specific T cells. The IC50_48h_ of panobinostat and vorinostat were 0.16 nM and 1.021 μM, respectively (Figure 1E,F).

Taken together, pharmacological HDAC inhibition was toxic towards STS cell lines as well as NY-ESO-1-specific T cells. In addition, panobinostat displayed higher cytotoxicity with IC50 values in lower concentrations when compared to vorinostat.

### 3.2. Panobinostat and Vorinostat Increases NY-ESO-1 Expression of SW982 Cells

To determine whether panobinostat or vorinostat could exert an alteration in NY-ESO-1 expression in SW982 and Fuji cells, we performed qRT-PCR and Western blotting. To use sub-lethal doses of HDACi for NY-ESO-1 modulation, we chose 0.005 μM panobinostat and 0.5 μM vorinostat to treat SW982 cells. The target cells were incubated with HDACi for 48 h.

**Figure 2:**
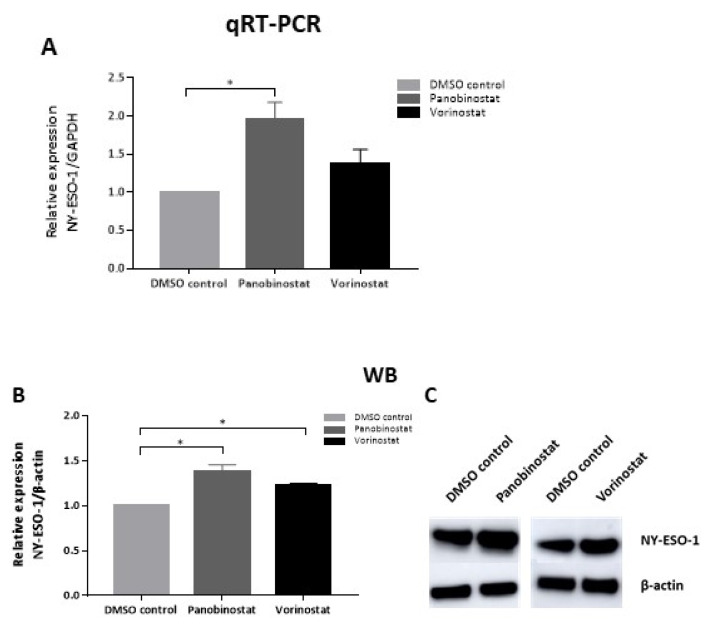
NY-ESO-1 expression in SW982 cells after treatment with panobinostat or vorinostat. SW982 cells were treated with 0.005 μM panobinostat or 0.5 μM vorinostat for 48 h, respectively. DMSO served as solvent control. The mRNA (**A**) and protein expression (**B**) of NY-ESO-1 were normalized to GAPDH and β-actin, respectively. Representative protein bands are displayed (**C**). Each column represents three individual experiments with three replicates. Statistical significance was calculated using one-way ANOVA. * *p* < 0.05.

Pronounced up-regulation of NY-ESO-1 at mRNA level was observed in SW982 cells after a 48h treatment with 0.005 μM panobinostat (panobinostat/DMSO: 1.96 ± 0.13 fold, *p* < 0.001, Figure 2A). Additionally, 0.5 μM vorinostat only showed a trend towards increased NY-ESO-1 expression at the mRNA level (vorinostat/DMSO: 1.37 ± 0.11 fold, *p* = 0.06, Figure 2A). No induction of NY-ESO-1 mRNA by HDACi was detected in NY-ESO-1-negative Fuji cells (data not shown).

Next, we examined the NY-ESO-1 expression at protein level using Western blot (WB). SW982 cells were treated as described above. Both 0.005 μM of panobinostat and 0.5 μM of vorinostat could significantly increase NY-ESO-1 levels (panobinostat/DMSO: 1.38 ± 0.08 fold, *p* < 0.001, Figure 2B; vorinostat/DMSO: 1.22 ± 0.03 fold, *p* < 0.01, Figure 2B). Figure 2C shows representative protein bands from the Western blot experiments. No induction of NY-ESO-1 protein could be observed by HDACi in NY-ESO-1- negative Fuji cells (data not shown).

### 3.3. Lysis of SW982 Cells Is Enhanced after Panobinostat or Vorinostat Pretreatment

**Figure 3:**
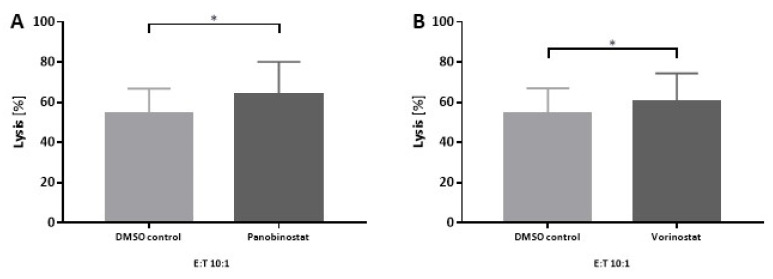
Lysis of SW982 cells by NY-ESO-1-specific T cells after 48 h pretreatment with panobinostat (**A**) or vorinostat (**B**). An effector (NY-ESO-1-specific T cells) to target (SW982 cells) ratio of 10:1 was used. DMSO pretreated SW982 cells served as solvent control. Data analyzed contain three individual experiments with three replicates. Statistical significance was calculated using a paired two-way student *t*-test. * *p* < 0.05.

Next, we determined whether the up-regulation of NY-ESO-1 at mRNA and protein levels in SW982 cells could increase the lysis by NY-ESO-1-specific T cells using a 12 h ^51^Cr release assay. SW982 cells were treated with a sublethal dose of HDACi (0.005 μM panobinostat, 0.5 μM vorinostat) for 48 h prior to the addition of NY-ESO-1-specific T cells. An effector to target cell (E:T) ratio of 10:1 was chosen according to previous experience with NY-ESO-1-specific T cells achieving a relevant rate of specific cell killing of SW982 cells [19,20]. A significant increase in lysis was achieved in HDACi pretreated SW982 cells. Pretreatment with panobinostat (0.005 μM) increased the lysis of SW982 cells by 10% (DMSO vs. panobinostat: 54% ± 13% vs. 64% ± 16%, *p* < 0.05, Figure 3A), whereas pretreatment with vorinostat (0.5 μM) increased cell killing by 6% (DMSO vs. vorinostat: 54% ± 13% vs. 60% ± 14%, *p* < 0.05, Figure 3B).

### 3.4. Panobinostat and Vorinostat Alter Surface Marker Expression on SW982 Cells

**Figure 4:**
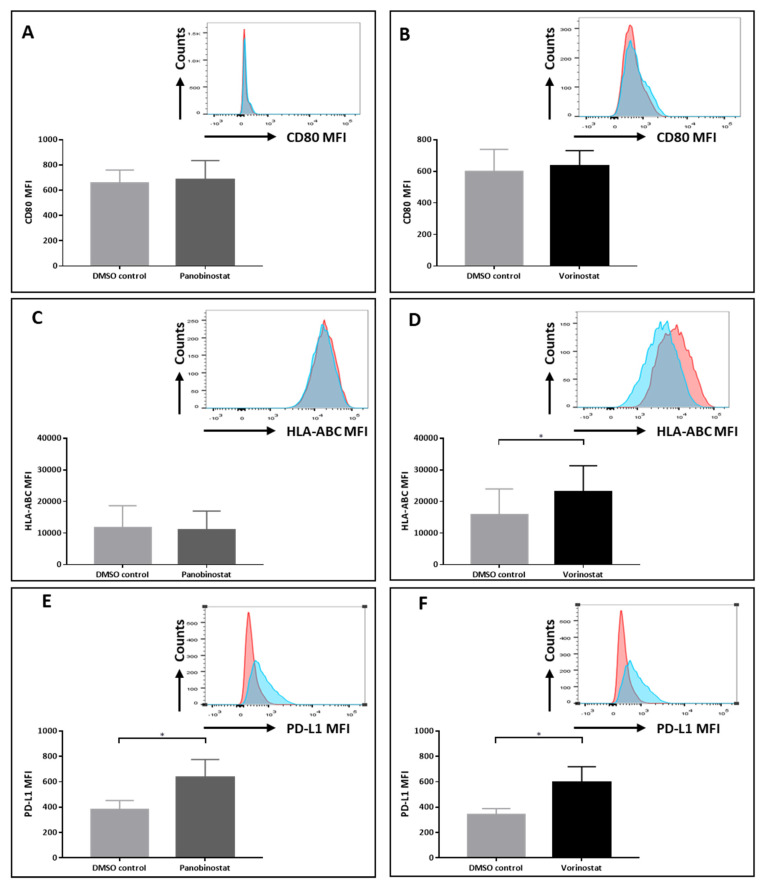
Alterations of surface markers on SW982 cells pretreated after HDACi for 48 h. The MFIs of CD80 (**A**,**B**), HLA-ABC (**C**,**D**) and PD-L1 (**E**,**F**) expression on panobinostat or vorinostat pretreated SW982 cells are displayed. DMSO served as solvent control. Histograms are showing representative flow cytometry results. Data analyzed contain at least four individual experiments with duplicates. Statistical significance was calculated using a paired two-way student *t* test. * *p* < 0.05.

To further characterize the influence of HDACi on sarcoma cells, we evaluated changes in the expression of surface markers including CD80, HLA-ABC and PD-L1 on SW982 cells. CD80 and PD-L1 are important immune checkpoint molecules and the MHC class I molecules HLA-ABC are crucial for antigen recognition by cytotoxic T cells. SW982 cells were treated with 0.005 μM panobinostat or 0.5 μM vorinostat for 48 h. Equivalent expression of CD80 could be observed between HDACi pretreated sarcoma cells and the DMSO control (Figure 4A,B). Whereas 0.005 μM panobinostat seemed to have no influences on HLA-ABC expression (Figure 4C), 0.5 μM vorinostat significantly increased HLA-ABC levels on SW982 cells when compared to the DMSO control (mean fluorescence intensity (MFI) DMSO vs. vorinostat: 15,860 ± 8117 vs. 22,999 ± 8332, *p* < 0.05; Figure 4D). Furthermore, a pronounced enhancement of PD-L1 expression could be observed after treatment with either 0.005 μM panobinostat (MFI DMSO vs. panobinostat: 380 ± 77 vs. 629 ± 140, *p* < 0.0001, Figure 4E) or 0.5 μM vorinostat (MFI DMSO vs. vorinostat: 339 ± 77 vs. 597 ± 121, *p* < 0.01, Figure 4F).

### 3.5. Panobinostat Upregulates Activation Maker on NY-ESO-1-Specific T Cells

**Figure 5:**
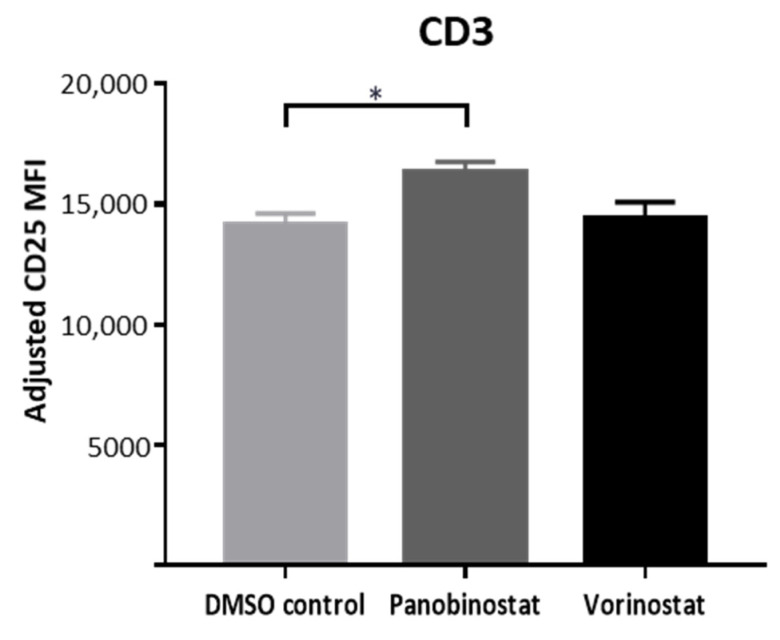
CD25 expression on NY-ESO-1-specific T cells after co-culture with panobinostat or vorinostat pretreated SW982 cells. DMSO served as solvent control. Data analyzed contain three individual experiments. Each experiment was performed in duplicates. Statistical significance was calculated using one-way ANOVA. * *p* < 0.05.

In an attempt to better understand the activation of NY-ESO-1-specific T cells, the expression of CD25 (interleukin-2 receptor alpha chain) on NY-ESO-1-specific T cells was determined after 48 h co-culture with panobinostat (0.005 μM) or vorinostat (0.5 μM) pretreated SW982 cells. Here, we adjusted the MFI to indicate CD25 expression, calculated as follows: MFI = CD25% × CD25 MFI (activated NY-ESO-1 T cells)—CD25% × CD25 MFI (inactivated NY-ESO-1 T cells). An increase in CD25 expression on all CD3+ NY-ESO-1-specific T cells activated by panobinostat pretreated SW982 cells was observed when compared to T cells activated by SW982 cells treated with the DMSO control (DMSO vs. panobinostat: 14,862 ± 455 vs. 17,014 ± 449, *p* = 0.047). In contrast, pretreatment of SW982 cells with vorinostat had no influence on CD25 expression (Figure 5).

### 3.6. Vorinostat Augments Cytokine Production in NY-ESO-1-Specific T Cells and Multifunctional NY-ESO-1-Specific T Cells

**Figure 6:**
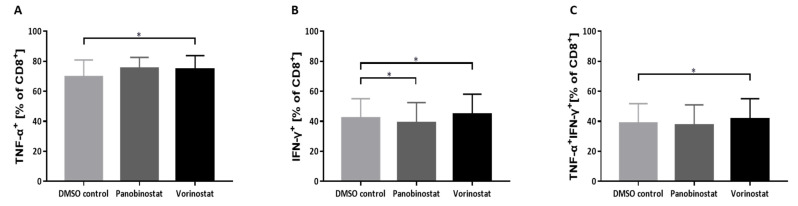
Intracellular cytokine production of NY-ESO-1-specific T cells after co-culture with HDACi pretreated SW982 cells. Production of TNF-α (**A**), IFN-γ release (**B**) and both cytokines (**C**) in NY-ESO-1-specific CD8+ T cells was determined after 6 h stimulation with panobinostat or vorinostat pretreated SW982 cells. DMSO served as solvent control. Six different individual donors were analyzed. Each experiment was performed in triplicates. Statistical significance was calculated using one-way ANOVA. * *p* < 0.05.

To further explore the effect of pretreated SW982 cells on NY-ESO-1-specific T cells, the quality of NY-ESO-1-specific T cells in terms of cytokine release and multifunctionality were evaluated. NY-ESO-1-specific T cells were generated from six different HDs and stimulated by panobinostat or vorinostat pretreated SW982 cells. CD8+ NY-ESO-1-specific T cells released more TNF-α (DMSO vs. vorinostat: % of CD8+, 69.52 ± 11.32 vs. 74.62 ± 9.21, *p* < 0.05; Figure 6A) and IFN-γ (DMSO vs. vorinostat: % of CD8+, 42.12 ± 13.03 vs. 44.50 ± 13.62, *p* < 0.01; Figure 6B) after stimulation with vorinostat pretreated SW982 cells. The frequency of multifunctional CD8+ NY-ESO-1-specific T cells in terms of TNF-α+IFN-γ+ were also elevated when stimulated with vorinostat pretreated SW982 cells (DMSO vs. panobinostat: 38.66 ± 13.16 vs. 41.65 ± 13.48, *p* < 0.01; Figure 6C). Under the stimulation of panobinostat pretreated SW982 cells, CD8+ NY-ESO-1-specific T cells released similar levels of TNF-α (DMSO vs. panobinostat: % of CD8+, 69.52 ± 11.32 vs. 75.37 ± 7.28, *p* = 0.11; Figure 6A) but secreted less IFN-γ (DMSO vs. panobinostat: % of CD8+, 42.12 ± 13.03 vs. 38.84 ± 13.71, *p* < 0.01; Figure 6B). The frequency of multifunctional CD8+ NY-ESO-1-specific T cells was not significantly influenced by panobinostat (DMSO vs. panobinostat: 38.66 ± 13.16 vs. 37.54 ± 13.52, *p* = 0.43; Figure 6C).

## 4. Discussion

STS are a heterogeneous group of rare solid tumors. Certain subtypes, such as synovial sarcoma and myxoid liposarcoma, are characterized by high expression of the CTA NY-ESO-1 [5,23]. Significant tumor regression with NY-ESO-1-specific T cells could be achieved in a variety of NY-ESO-1+ malignancies, including metastatic STS [8,10]. Since ACT with NY-ESO-1-specific T cells often displayed only transient tumor control [10], improved immunotherapeutic approaches are needed.

Ramachandran et al. showed in their clinical trial applying NY-ESO-1-speific T cells that patients with low NY-ESO-1 antigen expression had a shorter median duration of response when compared to the patients with high NY-ESO-1 antigen expression (low vs. high: 10 weeks vs. 30.9 weeks) [9]. Therefore, an option for further optimization of NY-ESO-1-directed T cell immunotherapy may be the induction of increased target antigen expression. Keung and Tawbi have pointed out the potential of epigenetic modulators to enhance antigen release and presentation. This may overcome the lack of antigen-driven T cell responses in STS [24]. Therefore, one approach to improve the clinical outcome of patients with STS might be the combination of HDACis with NY-ESO-1-specific T cells. In this study, the influences of two clinically approved pan HDACis (panobinostat and vorinostat) on NY-ESO-1 expression and the anti-tumor activity of NY-ESO-1-specific T cells were evaluated in STS.

In line with previous studies [25], our data demonstrate that HDACis exert strong anti-tumor activity against STS. Both panobinostat and vorinostat influenced the viability of STS cells in a dose-dependent manner.

Epigenetic targeting using HDACi have shown promising results in clinical trials for the treatment different malignancies [26,27,28]. It has been hypothesized that the accumulation of acetylated proteins using HDACi leads to a restoration or up-regulation of genes that have been epigenetically silenced in cancer cells. Cartron et al. reported that HDAC1, DNA methyltransferases Dnmt1 and Dnmt3b are involved in the negative regulation of NY-ESO-1. They showed that the HDACi valproic acid (VPA) together with the demethylating agent 5-aza-2-deoxycytidine (5aza) could increase NY-ESO-1 mRNA expression in glioma. In contrast, VPA alone was not able to promote NY-ESO-1 mRNA expression in glioma [14]. Furthermore, Bensaid et al. demonstrated that six different HDACis including panobinostat increased the mRNA expression of NY-ESO-1 induced by decitabine in pleural mesothelioma cells [29], whereas monotherapy with HDACis did not achieve this effect. Overall, the modulatory activity of HDACi on NY-ESO-1 expression in STS has not been well characterized. Different from the above studies, our data have demonstrated that panobinostat (0.005 μM) or vorinostat (0.5 μM) alone can increase NY-ESO-1 expression at the mRNA and protein level in STS, indicating the possible enhancement of immunogenicity and reduction of ITH.

A hallmark of cancer biology is ITH. Not all cells within a tumor express the same level of TAAs. McGranahan et al. reported in their study that tumors with both high clonal neoantigen burden and low neoantigen ITH were associated with significantly longer progression-free survival (PFS) in non-small cell lung cancer (NSCLC) and significantly improved OS in melanoma, respectively [12]. Thus, the modulation of NY-ESO-1 expression and the associated reduction in ITH may increase immunogenicity and thereby be favorable for cancer immunotherapy.

Previous studies have shown that panobinostat treatment resulted in the up-regulation of CD80 and MHC class I in melanoma cells [30]. In our study, CD80 expression was not affected by panobinostat or vorinostat treatment. In contrast, HLA-ABC expression was augmented after vorinostat treatment. In addition, both panobinostat and vorinostat significantly up-regulated PD-L1 expression on STS cells. The binding of PD-L1 with its ligand PD-1 on T cells can counteract the anti-tumor effect by inhibiting T cell proliferation, cytokine release and cytotoxic activity [31]. Of note, several studies have indeed demonstrated that PD-L1 expression could be increased in tumor cells following HDACi treatment [32,33], while accumulating studies have shown that blockade of PD-1/PD-L1 ligation promotes anti-tumor T cell activity [33,34]. Therefore, the combining of PD-1/PD-L1 blockade with the epigenetic modulation of NY-ESO-1 expression may reverse the unfavorable adverse effects on anti-tumor T cell responses.

CD25, also known as IL-2 receptor alpha chain (IL-2RA), is a surface molecule expressed on activated T cells, B cells, monocytes and macrophages. It is well known that CD25 is important for proliferation and in vivo anti-tumor activity of CD8+ T cells [35]. Our results indicate that panobinostat amplified CD25 expression on CD3+ NY-ESO-1-specific T cells. This is in line with previous reports showing that panobinostat can significantly increase the proportion of CD25+ tumor-specific T cells as well as the intensity of CD25 expression on tumor-specific T cells [36]. In our study, pretreatment of STS cells with HDACi resulted in increased lysis of tumor cells by NY-ESO-1-specific T cells. Reasons for this increased lysis may not only include the induction of higher target antigen expression but also increased HLA-ABC expression on STS cells as well as increased CD25 expression on NY-ESO-1-specific T cells.

Moreover, the pretreatment of tumor cells could also facilitate cytokine release and multifunctionality of T cells, which is highly associated with clinical outcomes [37]. Bae et al. delineated in their study that ACY241, an HDAC6 inhibitor, up-regulated anti-tumor activity of antigen-specific cytotoxic T lymphocyte against MM cells by increasing CD107a, IFN-γ, TNF-α and IL2 production [38]. Furthermore, Yang et al. demonstrated that the quality of CD8+ CD19 CAR-T cells in terms of the production of granzyme and cytokine release was enhanced by BH3 mimetics in their study, and this might be due to the up-regulated target antigen CD19 on tumor cells by BH3 [39]. These findings are substantiated by our own data, showing increased secretion of TNF-α and IFN-γ in NY-ESO-1-specific CD8+ T cells and a higher percentage of multifunctional NY-ESO-1-specific CD8+ T cells following the stimulation by HDACi pretreated STS cells.

In summary, the current study shows immunomodulatory activities of the clinically approved HDACis panobinostat and vorinostat to enhance the anti-tumor efficacy of NY-ESO-1-specific T cells on NY-ESO-1-positive STS. Even though the impact on each aspect mediated by HDACi, including NY-ESO-1 target expression, HLA-ABC expression, T cell activation and T-cell-mediated cytotoxicity, was relatively low, all improvements, together, might have the potential to be an important part of increased activity by NY-ESO-1-specific T cells. Importantly, sublethal doses of HDACis were used in this study to allow for downstream experiments, and higher doses might further increase the benefit of combination treatment. Overall, the combination of HDACis with ACT might represent a promising approach to improve cellular cancer immunotherapy and, therefore, warrants further preclinical and clinical investigation.

## Data Availability

Not applicable.
